# Rye (*Secale cereale* L.) revisited—nutritional composition, functional benefits, and role in sustainable diets

**DOI:** 10.3389/fnut.2025.1666455

**Published:** 2025-10-27

**Authors:** Daiva Zadeike, Claire Copperstone, Olha Aleksandrova, Derya Özalp Ünal, Katarina Šavikin, Jelena Živković, Mustafa Güzel, Hatice Kalkan Yildirim, Ibrahim Ender Künili, Teodora Ivanova, Özge Özmen, Filippos Bantis, Jelena Milešević, Bálint Balázs, Sónia Negrão, Marija Knez

**Affiliations:** ^1^Department of Food Science and Technology, Faculty of Chemical Technology, Kaunas University of Technology, Kaunas, Lithuania; ^2^Department of Food Science, Nutrition and Dietetics, Faculty of Health Sciences, University of Malta, Msida, Malta; ^3^Chair of Rural Economy and Economics, Institute of Agricultural and Environmental Sciences, Estonian University of Life Sciences, Tartu, Estonia; ^4^Department of Quality Control, Field Crops Central Research Institute, Ankara, Türkiye; ^5^Department for Pharmaceutical Research and Development, Institute for Medicinal Plants Research “Dr Josif Pančić”, Belgrade, Serbia; ^6^Department of Food Engineering, Faculty of Engineering, Hitit University, Corum, Türkiye; ^7^Department of Food Engineering, Faculty of Engineering, Ege University, Bornova, İzmir, Türkiye; ^8^Department of Fishing and Fish Processing Technology, Faculty of Marine Sciences and Technology, Çanakkale Onsekiz Mart University, Çanakkale, Türkiye; ^9^Department of Plant and Fungal Diversity and Resources, Institute of Biodiversity and Ecosystem Research - Bulgarian Academy of Sciences, Sofia, Bulgaria; ^10^Department of Food Engineering, Faculty of Engineering, Adana Alparslan Türkeş Science and Technology University, Adana, Türkiye; ^11^Department of Agriculture, University of Western Macedonia, Flórina, Greece; ^12^Centre of Research Excellence in Nutrition and Metabolism, Institute for Medical Research, National Institute of Republic of Serbia, University of Belgrade, Belgrade, Serbia; ^13^Environmental Social Science Research Group (ESSRG) Nonprofit Kft, Budapest, Hungary; ^14^School of Biology and Environmental Science, University College Dublin, Dublin, Ireland

**Keywords:** rye, wholegrain consumption, nutrition composition, health benefits, non-communicable diseases, sustainability, environment

## Abstract

Rye (*Secale cereale* L.) is increasingly recognized as a sustainable cereal with significant nutritional, ecological, and economic potential. While previous studies have highlighted its dietary fiber (DF), bioactive compounds, and associated health benefits, this review provides an updated synthesis that integrates recent findings on rye's role in human health, food security, and sustainability. In particular, it emphasizes novel evidence on rye's functional properties, its potential contributions to plant-based dietary strategies, and its economic and social relevance. By consolidating current knowledge and outlining future directions for product development and dietary innovation, this work offers a fresh perspective that extends beyond earlier 0 reviews focused on rye.

## 1 Introduction

Plant-based nutrition is increasingly recognized as an effective long-term strategy for addressing both health and environmental challenges (191). In this context, rye emerges as a resilient and sustainable crop offering significant dietary, environmental, and economic advantages (1).

Rye (*Secale cereale* L.) is one of the oldest and most resilient cereal grains in Europe, playing a vital role in both traditional agriculture and food culture (2). Rye is particularly valued for its ability to grow in poor soils and cold climates, which makes it an essential crop in many European regions. Traditionally, rye has been used to produce rye bread, a dense, nutritious bakery product widely consumed in countries such as Germany, Poland, and throughout the Scandinavian and Baltic regions. Beyond human consumption, rye grain also serves as an important component of animal feed, supporting the livestock industry (3).

**Figure 1:**
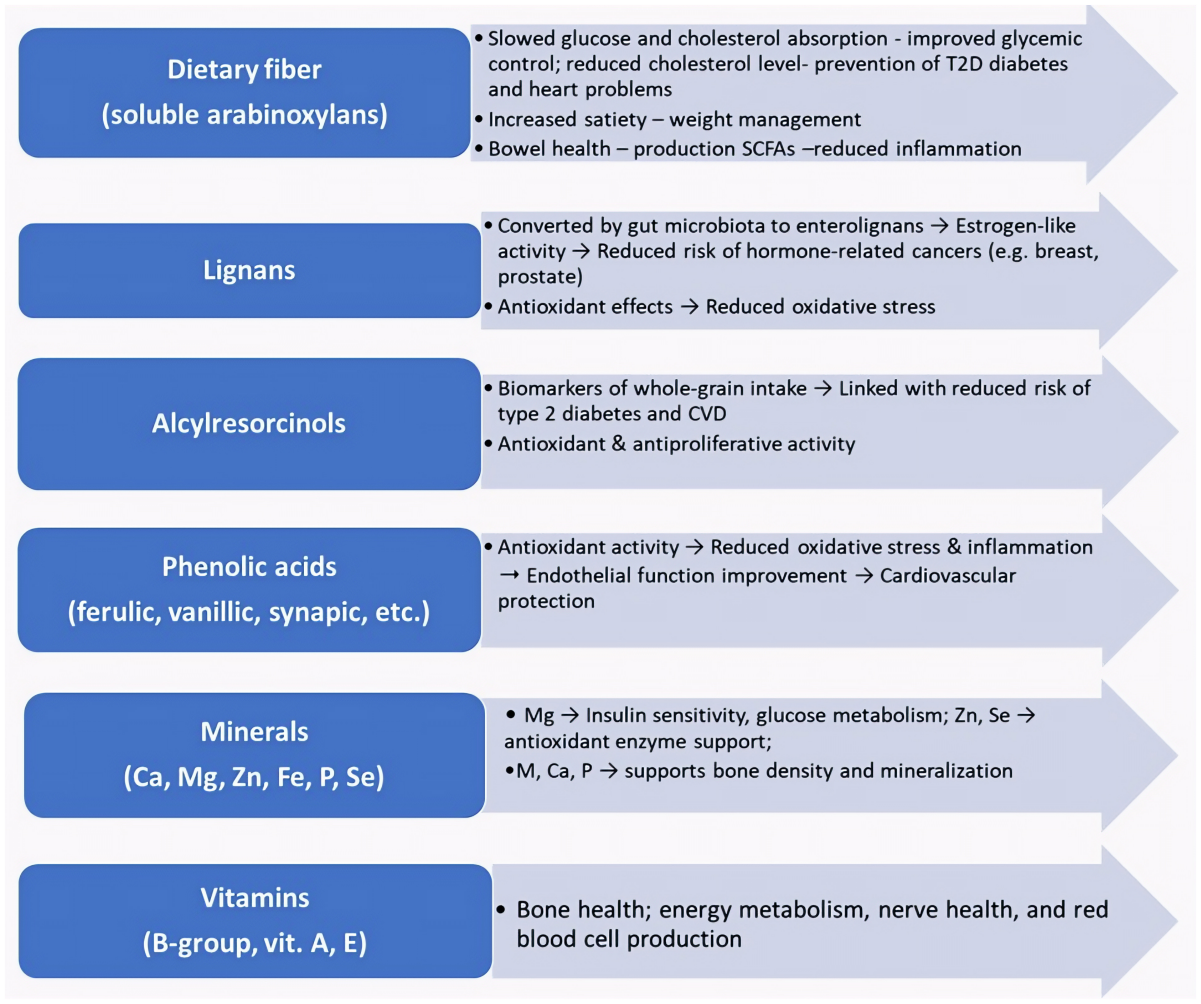
Rye bioactive compounds and their health outcomes.

Rye is a rich source of proteins, starch, and bioactive compounds, such as dietary fiber, antioxidants, and essential micronutrients (Figure 1). Whole-grain rye contains a high level of dietary fiber (DF), which supports gastrointestinal health through antioxidant and anti-inflammatory phytochemicals (4). In addition to its fiber content, rye grains contain a wide spectrum of bioactive compounds, including alkylresorcinols, ferulic acid, catechol, sinapic acid, vanillin, and vanillic acid, that exhibit antioxidant properties and may support immune function and mitigate age-associated physiological decline (5, 45). The micronutrient composition of rye is considered equally vital to its macronutrient content, playing a critical role in regulating numerous biochemical processes within the human body. The inclusion of rye in a nutrient-dense, balanced diet may contribute to the prevention of various chronic diseases. Notably, rye naturally contains a distinctive profile of essential vitamins and minerals (6). Moreover, the synergistic consumption of leguminous and cereal crops ensures a complete amino acid profile, optimizing dietary balance. This integrative nutritional approach addresses potential dietary deficiencies while supporting sustainable protein consumption strategies (7).

The European sustainability trends in agriculture may lead to an increasing interest in rye cultivation and consumption (8). The ability of rye to grow in poor conditions makes it a valuable crop for farmers, as it can be integrated into crop rotation systems to improve soil quality, ensuring biodiversity protection through efficiently managed resources, which supports sustainable farming (9, 10).

This review presents a thorough and current examination of the nutritional properties and health benefits of rye, distinguishing it from other prominent agricultural crops. It provides a fresh synthesis of the latest research, while also delving into rye's vital role in enhancing global food security and promoting sustainable farming practices. By integrating current research on rye's economic and social impacts, this review identifies emerging dietary trends and potential avenues for innovative product development.

## 2 Cultivation and consumption of rye across Europe

The European Union (EU) harvested 7.8 million tons of rye in 2023, a very similar quantity to that in 2022 (11). According to The Food and Agriculture Organization's global statistical database (FAOSTAT) (12), the top rye-producing countries in the EU were Germany (3.13 M tons) and Poland (2.4 M tons), followed by Denmark (883.5 K tons), Belarus (800 K tons), and Ukraine (334.6 K tons). Germany's production, which represents two-fifths of the EU's total output, saw a slight decline of 0.3%, contributing to the overall stagnation across the EU.

Elsewhere, the steep declines in rye production in Spain (−46.6%) and Denmark (−13.1%) were largely countered by the increases in Poland (up to 5.4%), Hungary (up to 59.1%), and Finland (up to 41.3%). The global rye market size was valued at USD 3.89 billion in 2023 and is expected to grow at a compound annual growth rate (CAGR) of 3.6% from 2024 to 2030 (11).

Although wheat dominates in many European countries, rye remains essential due to its adaptability to poor soils and cooler climates, making it suitable for a wide range of regions. This resilience makes rye valuable for farmers, especially as it supports crop rotation, improves soil quality, and prevents erosion (9). While increasing yield is a key aspect of rye cultivation, this health-promoting cereal also offers important sustainability benefits for food and feed production. Rye is a resilient crop that thrives in poor soils and harsh climates, reducing the need for intensive irrigation and chemical inputs while improving soil health through its extensive root system and role as a cover crop (9**?** , 10). Additionally, its high dietary fiber content supports livestock gut health, while its natural pest resistance promotes eco-friendly farming—making rye a sustainable choice for food and feed (5).

Germany is the largest producer of rye in Europe, and rye is deeply ingrained in the country's culinary culture. Rye bread (Roggenbrot) is a staple in German households, and the country is known for its diverse rye-based products. Occupying 28.7% of the total bread consumption (58.9 kg/year per capita), brown bread is preferred in Germany, followed by toast bread (21.4%) and seeded bread or cornbread (15.5%) (14). Rye has long been a traditional crop in Lithuania and Poland, with rye bread being a main element of the cuisine (15). The most renowned national heritage of Lithuania is dark rye bread, traditionally made from wholemeal rye flour. French rye is primarily used in the production of bread and rye-based products. In countries such as Denmark and Finland, where wholemeal rye bread is the most widely consumed, around 40% of the dietary fiber comes from rye-based products (16). Rye bread is the main source of whole-grain intake, contributing 58% in children and 64% in adults (17). While wheat is the primary cereal crop in France, rye has long been an important crop in the northern regions of the country, where the cooler climate allows for rye cultivation. In other European countries, rye consumption is moderate to low but still prevalent, particularly in Austria, the Czech Republic, Slovakia, and Latvia, where it is featured in traditional breads and baked goods (14). These trends highlight the cultural and regional differences in rye consumption across Europe, showcasing its persistent importance in traditional diets and its potential role in promoting sustainable and health-conscious eating habits.

## 3 Rye nutritional quality

**Figure 2:**
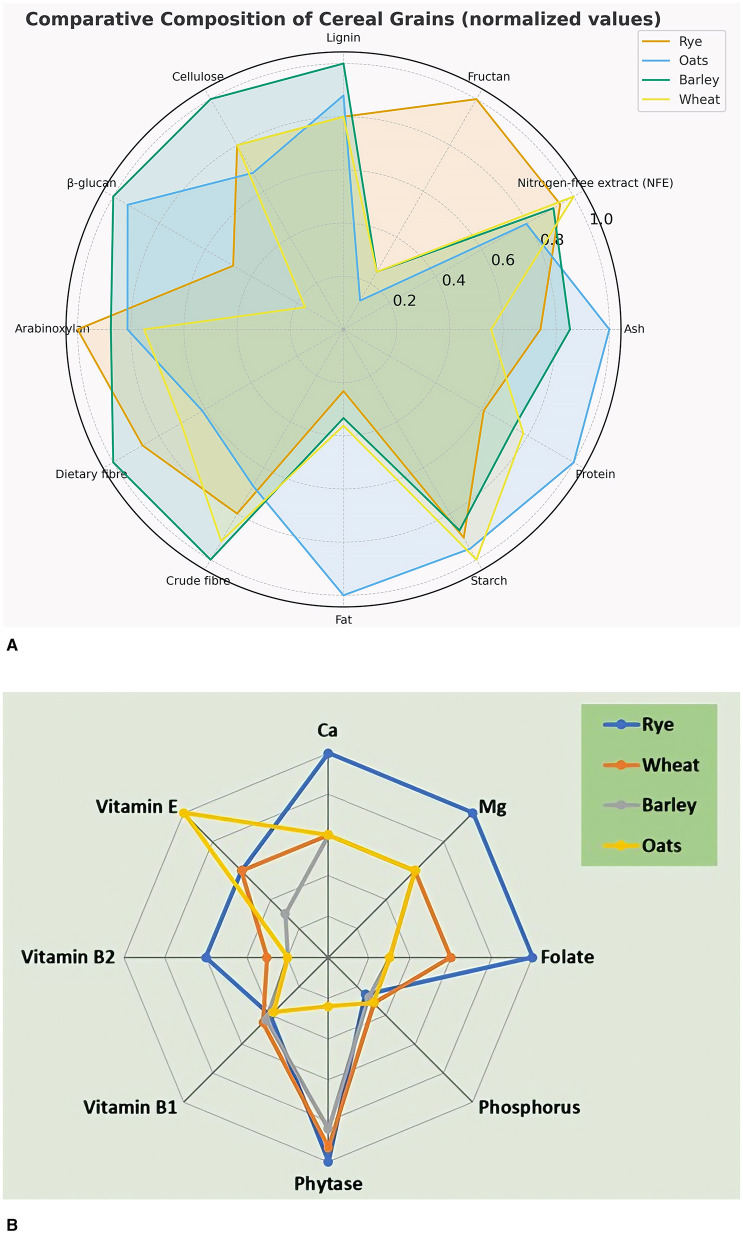
**(A, B)** Nutritional components of rye compared to wheat, barley, and oats.

Rye (*S. cereale L*.), a member of the Poaceae family and genetically related to wheat and barley, is widely recognized for its nutritional value (18). Compared to other cereals, rye demonstrates superior nutritional value, providing higher levels of dietary fiber, antioxidants, health-promoting phytochemicals, and essential macro- and micronutrients (Figure 2). Additionally, rye exhibits greater resistance to diseases and various pathogenic stresses (19). Due to its richness in nutrients and bioactive compounds, rye is widely used in the food industry, second only to wheat, for making bread, biscuits, and flakes (20).

### 3.1 Rye macronutrients

Rye fiber supports weight regulation and promotes digestive health (21). Rye grain has higher fiber content than other cereals (22) (Table 1).

**Table 1:** Macronutrient composition and nutritional properties of rye compared to other cereals.

**Component**	**Rye grain**	**Comparison with other cereals**	**References**
Dietary fiber (DF)	19.9% TDF; 13–17% of whole grain; ~20% soluble. Main components: AX (8.0–12.1%), fructans (4.5–6.6%), β-glucans (1.3–2.2%).	Higher than barley (15.2%) and wheat (13.5%); rice (2.7–9.9%). More soluble AX compared to wheat	(22, 23, 182)
Arabinoxylans (AX)	~64% of rye DF; high water-binding capacity	Higher solubility and quantity than in wheat	(23, 24)
fructans	Rich source; prebiotic, supports gut microbiota, regulates glucose and lipid metabolism.	Unique among cereals	(26–28)
β-Glucan	Soluble DF lowers blood glucose, insulin, and cholesterol.	Similar components in barley and oats, but lower concentration	(25)
Protein	8–15% (average ~11% dry weight); varies by genotype and conditions. Main proteins: albumins (29–40%), globulins (8–11%), prolamins (17–19%), glutelins (9–15%).	Lower than wheat (17.8%), barley (17.3%), oat (13%), and maize (10.4%)	(2, 20, 23, 55)
Amino acids	Higher lysine, proline, and glutamine than wheat; limited in tryptophan and isoleucine.	Lysine: rye (3.49 g/kg) > wheat (3.22 g/kg) > triticale (3.01 g/kg). Still a limiting amino acid in cereals	(30, 31)
Starch	55–65%	Lower than wheat (63–72%), higher than barley (50–64%).	(2, 32)
Lipids	2–3%; high in polyunsaturated fatty acids.	Comparable to oat; higher than buckwheat (1.8%), barley (1.5%), and wheat (1.2%).	(2)
Fatty acids (FA)	Unsaturated FA (81.46%); linoleic acid (18.9–59.3%).	Slightly higher UFA than oat (80.12%), triticale (79.51%), wheat, and barley.	(34, 35)

The main components of rye fiber are arabinoxylans, fructans, and β-glucans, which have a structure similar to wheat but a higher percentage of soluble AX (23). Arabinoxylans bind water effectively, which is beneficial for digestion (24). β-Glucan, a soluble fiber, provides health benefits by moderating blood glucose, insulin, and cholesterol levels (25). Rye is also rich in fructans, which exhibit distinct functional properties relative to other cereals (26). Fructans serve as a primary carbon source for bifidobacteria, supporting gut health and protecting against pathogens (27). As a prebiotic, fructans improve glucose regulation and lipid metabolism, and reduce lipopolysaccharide levels (28).

The macronutrient composition and key nutritional properties of rye in comparison to other common cereals are presented in Table 1. The protein content in rye kernels varies depending on the genotype and growing conditions (2). Rye contains less protein on average than wheat, barley, and oats (29).

Albumins are the main protein fraction, followed by globulins, prolamins, and glutelins (20). Compared to wheat, rye proteins offer a slightly better amino acid profile with higher levels of lysine, proline, and glutamine, although they remain limited in tryptophan and isoleucine (30). Rye is notable for its relatively high lysine content compared to wheat and triticale, although lysine is still the most limiting amino acid in rye and other cereals (31). The starch content in rye grain is lower than in wheat but higher than in barley (2, 32). Rye lipids, rich in polyunsaturated fatty acids, contribute to health benefits and protect against chronic diseases such as cardiovascular issues, neurological disorders, cancer, inflammation, obesity, and diabetes (33). Rye's lipid content is similar to that of oat, slightly higher than buckwheat, barley, and wheat (2). Rye also contains more unsaturated fatty acids than oats, triticale, durum and common wheat, and barley-linoleic acid being the dominant type (34, 35).

### 3.2 Rye micronutrients

Rye naturally contains a distinctive composition of micronutrients that support numerous biochemical processes in the human body (36). While other cereals may be richer in some minerals, rye stands out for its high dietary fiber content and wide range of vitamins and bioactive compounds (35), making it a valuable component of a healthy diet, especially in comparison with whole wheat (Figure 2).

Notably, rye has the highest phytase activity among oats, barley, and wheat, meaning it has the greatest potential to break down phytates. Compared to the other major food crops, oats have a relatively high phytate content. All cereal grains have significant amounts of phytate, but the lowest content of the phytate-cleaving enzyme, phytase, is in oats compared to wheat, barley, and rye (37). Rye genotypes also exhibit higher levels of Ca and Mg compared to triticale (38). Rye flour provides significant amounts of folate, which is recognized for its role in preventing megaloblastic anemia and reducing the risk of neural tube defects during pregnancy (39).

Table 2 provides a detailed comparison of the micronutrient content of rye with that of other cereals, highlighting its unique nutritional benefits. The main bioactive phytochemicals in rye are phenolic acids, phytosterols, alkylresorcinols, and lignans (16). Several other bioactive compounds, including flavonoids, anthocyanins, tocopherols, and tocotrienols, have also been identified in rye (5, 40, 41). Furthermore, rye is a good source of α-tocopherol similar to wheat; however, oats are characterized by the highest vitamin E content (2, 35).

**Table 2:** Rye micronutrients and nutritional properties compared to other cereals.

**Component**	**Rye grain**	**Comparison with other cereals**	**References**
Phytochemicals	Concentrated in the bran fraction; phenolic acids (~0.5–1.0 g/kg); Phytosterols (~0.7–1.4 g/kg); Alkylresorcinols (~0.7–1.2 g/kg), lignans (18–38 mg/kg)	Higher polyphenols (125–255 mg/100 g) than in barley (50–196 mg/100 g), oat (9–34 mg/100 g), wheat (70–145 mg/100 g), and rice (54–313 mg/100 g); higher alcylres (570–3,220 μg/g) than in wheat (200–750 μg/g), barley (150 μg/g), oat, and rice.	(16, 36, 78, 183)
Vitamins	Vitamin E; B-group vitamins: riboflavin, tocopherol, thiamine, B6, niacin, choline, and folate	Higher B-group vitamins than in wheat; Vitamin E lower than in oat; lower tocopherols (0.4–0.7 mg/100 g) than in barley (4.7–6.8 mg/100 g) and wheat (2.3–8.0 mg/100 g).	(2, 5, 39–41, 78)
Minerals	Fe, Zn, Mn, Cu, Ca, and Mg	Higher amounts than in wheat; higher Ca (0.02–0.03%) and Mg (0.13–0.14%) levels than in triticale	(35, 36, 38)

Plant-derived macronutrients and phytochemicals play an essential role in supporting a healthy lifestyle due to their nutritional and health-related benefits, including prebiotic effects on gut microbiota and antioxidant capabilities (5). By mitigating the damaging effects of free radicals and oxidative stress, they exhibit antioxidant and anti-inflammatory properties that promote both intestinal and overall systemic health (42).

## 4 Health benefits of rye

### 4.1 Digestive and cardiovascular health

Rye dietary fiber (DF) —notably arabinoxylans and β-glucans—slows gastric emptying, which may improve nutrient absorption and help maintain normal intestinal motility (42). Dietary metabolites act in concert with the gut microbiota to help support intestinal ecosystem balance. According to the literature, metabolite profiles from rye sourdough and *in vitro* colonic fermentation appear more favorable for intestinal health than those from other cereals (42).

In addition, rye fiber exhibits prebiotic effects—it can suppress pathogens and selectively promote beneficial bacteria (e.g., Lactobacillus, Bifidobacterium), which ferment fiber into short-chain fatty acids (SCFAs) that help regulate metabolic and immune processes (43, 44). Regular consumption of whole-grain rye can increase beneficial bacteria and promote a healthier gut microbiota, which is associated with improved metabolic and immune outcomes (45). The intestinal functionality of rye products may include increased fecal bulk, binding and efficient elimination of potentially toxic metabolites, and release of protective components such as lignans (46).

Currently, there is one EU-authorized health claim, based on a positive scientific opinion issued by the European Food Safety Authority (EFSA) Panel on Dietetic Products, Nutrition, and Allergies (47). This claim states that rye DF, when consumed in sufficient amounts, contributes to normal bowel function. Moreover, clinical evidence indicates that rye can help prevent constipation and improve bowel regularity, thereby reducing the need for laxatives, likely due to its high fiber content (48). Preclinical and clinical data suggest that incorporating alternative grains and dietary fiber into sourdough bread formulations can reduce risk factors for non-communicable diseases and beneficially modulate the gut microbiota (45, 49).

Beyond its digestive benefits, whole-grain rye consumption is also associated with cardiovascular benefits. Whole-grain rye has been associated with improved lipid profiles, lower blood pressure, and reduced inflammation—factors relevant to cardiovascular health (43). Elevated total and low-density lipoprotein (LDL) cholesterol are established risk factors for cardiovascular disease. Diets rich in whole grains are associated with a reduction in cholesterol levels compared to a refined-grain diet, and with a reduced risk of coronary heart disease (50).

Viscous, soluble DF has been shown to lower both systolic and diastolic blood pressure (51) and to exert more favorable effects on cardiometabolic risk factors (e.g., blood lipid levels, glycemic control) than non-viscous or insoluble fibers (52). One mechanism underlying fiber's cholesterol-lowering properties is bile-acid binding in the small intestine, which promotes their excretion (53). Additionally, alkylresorcinols-phenolic lipids abundant in wheat and rye may reduce cholesterol absorption, potentially enhancing rye's cholesterol-lowering effect (54).

Higher whole-grain consumption is also associated with lower body mass index and may reduce the prevalence of metabolic syndrome (MbS), which comprises hyperglycemia, dyslipidemia, hypertension, and obesity (55). These factors, alone or in combination, increase cardiovascular disease (CVD) risk (56, 57). Whole-grain-rich diets have been associated with a reduced incidence of CVD, largely via improvements in obesity and lipid profiles (58–60). Overall, the rye-based products may be particularly useful for elucidating the metabolic effects of rye consumption.

### 4.2 Diabetes control and weight management effects

The glycemic index (GI) indicates the extent to which a particular type of food raises blood glucose levels after eating (61). Blood sugar regulation is crucial in managing diabetes; dietary strategies include emphasizing low-glycemic index (GI) foods and high fiber, and reducing rapidly digestible carbohydrates (62). Studies report lower post-prandial glycemic responses when whole grains are from rye (63). Highly viscous rye soluble arabinoxylans (AX) resist digestion and may help to attenuate post-prandial glycemia and cholesterol levels (58, 64). Randomized controlled trials have indicated that medium-to-long-term whole-grain intake reduces fasting glucose concentration compared with refined-grain foods (65).

Reduced insulin sensitivity is a crucial contributor to the development and progression of type 2 diabetes mellitus (T2DM) (66). In obesity and T2DM, insulin resistance—a diminished response to insulin—is common (67). Replacing refined grains with whole grains leads to improvements in cardiometabolic biomarkers associated with cardiovascular disease risk (68). Using a metabolomics approach, one clinical study found a lower post-prandial insulin response after sourdough rye bread compared with wheat bread (69). Prospective cohorts have also reported a 27–30% lower risk of T2DM with higher whole-grain intake and a 28–37% lower risk with higher cereal fiber intake (70, 192). Collectively, these findings underscore the vital role of integrating whole-grain rye as a part of a balanced diet, given its potential to improve glycemic control and cardiometabolic markers. Rye-based foods (e.g., bread and porridges) have been reported to be more satiating than wheat-based products (71), which may aid weight management. Compared to wheat-based products, consumption of rye products is associated with lower body weight, likely due to their higher fiber content and increased satiety (21, 72). Weight gain was inversely associated with high-fiber whole-grain intake, supporting the role of whole grains in weight control (73). Some whole-grain cereals—especially wheat and rye—are good sources of dietary betaine, which has beneficial effects on obesity, alcohol-induced and metabolic-associated liver disease, diabetes, cardiovascular diseases, and certain cancers (74). A primary dietary source of betaine, cereal grains can provide more than 85% of daily intake (75). Higher betaine intake is associated with a lower risk of overweight and obesity (76).

### 4.3 Anti-inflammatory effects of rye and role in cancer prevention

Inflammatory reactions can promote the progression of certain chronic diseases, such as Alzheimer's disease, type 2 diabetes, and atherosclerosis (77). Certain phenolic compounds have shown potential in counteracting these conditions by modulating inflammatory pathways. Diets consisting of whole-grain cereals, compared with refined grains and their fractions, have been reported to influence plasma phytochemical levels and reduce oxidative stress and inflammation (45, 78). The antioxidant activity of polyphenols plays an important role in protecting against oxidative stress-induced neurodegenerative diseases, CVD, chronic oxidative cellular damage, viral and bacterial infections, diabetes, inflammatory disorders, and infectious illnesses (79, 80).

Regarding the anti-inflammatory effects of whole-grain diets, most studies focus on the health benefits of phenolic acids (PA) and their antioxidant properties. Most PAs in rye grain are in bound form, as in other cereals, with only 1–5% as free phenolic acids, of which ferulic acid is the most abundant (81). Water-soluble PAs, containing only 10–30% of the total content, exhibit most of the antioxidant activity (81). According to the literature, the content of phenolic compounds is 15- to 18-fold higher in rye bran than in the endosperm, which contains only 17% of the total phenolic content (82). PAs in rye grain possess anti-inflammatory effects by potentially reducing pro-inflammatory cytokines, acting as antioxidants to combat oxidative stress, and supporting overall health through mechanisms that may include beneficial interactions with the gut microbiota (81).

Lignans are less abundant phenolic compounds that are generally found in plant material in a bound form (83). Such bound rye phytochemicals have been reported to increase plasma total antioxidant capacity, which can directly reduce oxidative stress (84). It has been demonstrated that consumption of wholemeal rye bread results in a significant increase in plasma and urine enterolactone levels in healthy individuals compared with white wheat bread (85).

Whole-grain intake has been suggested to be beneficial in preventing several lifestyle-related chronic diseases, including certain types of cancer (73). An inverse association between the intake of whole-grain products and pancreatic cancer incidence was also reported by Lei et al. (86). Whole grains, rich in fiber and lignans, may help reduce the risk of hormone-related cancers, such as breast cancer (87). The phytoestrogenic properties of lignans show potential to slow down hormone-sensitive cancers, including breast, prostate, and colon cancer (46). The lignans in rye undergo bacterial conversion in the gut to produce compounds that may help reduce breast cancer risk by lowering estrogenic absorption (88) and may reduce the risk of developing bowel cancer by improving bowel function and decreasing the presence of certain compounds that increase colon cancer risk (89). Rye consumption may also lower the risk of bowel cancer by improving bowel function and decreasing carcinogenic compounds in the colon (89). Furthermore, high-fiber rye and wheat both increased fecal bulk. Still, only rye significantly increased fecal butyrate concentrations, which are important for maintaining healthy colonocytes and may act as anticancer agents (90).

Overall, findings from intake studies suggest that cereal phytochemicals provide only limited or modest protection against oxidative stress, indicating the need for further research to confirm and strengthen these observations.

### 4.4 Rye diet contribution to bone health

The growth and metabolism of bones depend on trace elements, which include iron, zinc, copper, calcium, phosphorus, and magnesium. Both deficiencies and excesses of these elements can increase the risk of bone diseases, including osteoporosis (91, 92).

Osteoporosis is a major global health issue. It is a systemic disease that reduces bone mass and quality, making bones fragile and prone to fractures. These fractures often lead to disability, lower quality of life, and higher mortality (13, 93). A review of 40 studies involving over 79,000 older adults from Asia, Europe, and America found that about 21.7% of them had osteoporosis (94).

Minerals, such as Ca, Mg, and P, are critical in supporting bone density and strength. Calcium is essential for the development, growth, and maintenance of bones (95), and magnesium participates in metabolic pathways in cells, stimulating the activity of osteoblasts and enzymes, involved in the bone formation process, and directly affects bone density (96). Phosphorus is the second most fundamental component of bone tissue after calcium, almost 85% of which is stored in bones and teeth (97). Its deficiency leads to defects in mineral deposition related to bone disorders, rickets, impaired growth, and disordered bone mineralization (98).

Nutritional strategies are key for preventing osteoporosis. Besides calcium, vitamin D, and protein (99), short-chain fatty acids (193), dietary fiber (100), and polyphenols and flavonoids (101) also contribute to building bone mass.

Recent research confirms that whole-grain diets improve bone health by increasing bone mineral density and balancing bone resorption and formation (194). Diets rich in milk, cereal, and whole grains are linked to higher bone mineral density (102). Overall, a healthy diet riche in whole grains may help prevent osteoporosis and lower the risk of fractures.

Rye may enhance bone health mainly due to its abundant mineral content, which includes Ca, Mg, K, Fe, Zn, Cu, and vitamins (B vitamins, vitamins E and A) (103) that are essential nutrients vital for sustaining bone density, strength, and proper mineralization. Rye stands out among cereals because of its higher Ca, Mg, and P content, which are crucial for bone mineralization and density, compared with wheat and oats, which contribute important minerals but provide less calcium (Table 3). Brown rice contributes some minerals but is weaker for bone health compared to rye, and white rice offers minimal benefit (104).

**Table 3:** Contribution of whole grains to bone health and osteoporosis prevention.

**Grain**	**Key nutrients**	**Specific benefits**	**Limitations compared to rye**	**References**
Rye	High in Ca, Mg, P; also contains Fe, Zn, Cu; B-group vitamins, A, E	Strongly supports bone density and mineralization; good balance of Ca, Mg, and P; functional food for lifelong skeletal health	Less commonly consumed than wheat or rice; gluten-containing (not suitable for celiac patients)	(36, 103)
Wheat	Good source of Mg, P, Zn, B-group vitamins, vitamin K	Supports bone metabolism and provides energy for growth; widely available	Lower Ca content than rye; mineral bioavailability may be reduced by phytates	(194)
Oats	Rich in Mg, P, Fe, Zn; also contains β-glucans (fiber)	Supports bone strength and metabolic activity; fiber has anti-inflammatory effects beneficial for bone health	Lower Ca content than rye; consumed more as a breakfast grain rather than a staple	(184)
Brown rice	Provides Mg, P, some B vitamins, and trace minerals	Staple food worldwide contributes to baseline mineral intake	Relatively low in Ca and Mg compared to rye; polished white rice loses most nutrients; weaker effect on bone density	(104)

The balanced mineral profile of rye supports bone development and maintenance, while also helping to prevent conditions such as osteoporosis and rickets. In addition, its mineral content contributes to the regulation of metabolic processes involved in bone formation and repair, making rye a valuable dietary component for sustaining skeletal health.

## 5 Antinutrients and toxins in rye and their reduction methods

### 5.1 Antinutritional factors and potential toxins in rye and rye products

Antinutritional (AN) factors are compounds naturally found in edible seeds that affect the bioavailability of nutrients, especially proteins, minerals, and vitamins, by binding to them (105). In this case, antinutritional factors may cause harmful effects on the growth and performance in humans and animals by disrupting the uptake and absorption of nutritious components (106). The main antinutritional substances in rye grain include pentosans, phytates, trypsin, and amylase inhibitors (107).

The most important cereal antinutrient is phytic acid (PA), the main storage form of phosphate, amounting to 70% of total seed phosphate content (108) (Table 4). PA was found in a range of 0.54–1.46 g/100 g and 0.19–0.43 g/100 g in rye and rye bread, respectively (109). PA has the ability to combine metal ions, especially Zn, Fe, and Ca, making them unavailable in humans due to very low intrinsic phytase activity in the digestive tract (41, 110).

**Table 4:** The levels of potential antinutrients in rye and their impact on health.

**Antinutrients**	**Typical level**	**Health impacts**	**References**
Phytic acid	Up to 540–1,460 μg/g d.w. whole meal; higher than in wheat (390–1,350 μg/g d.w.), oats (420–1,160 μg/g d.w.), and barley (380–1,120 μg/g d.w.)	↓ mineral bioavailability (Fe, Zn, Ca, and Mg), ↓ protein digestibility; antioxidant at moderate intake	(41, 108–110)
Arabinoxylans	6–10% DM (total), 2–3% DM soluble; content differs between rye hybrids and population cultivars	↑ digesta viscosity (↓ enzyme access); prebiotic	(185–187)
Enzyme inhibitors (Trypsin/amylase)	Low–moderate (bran-enriched); accumulate during grain development amylase-trypsin inhibitors (ATIs)	↓ protein/amidon digestibility; may alter glycemic response	(136, 138)
Phenolic acids and alkylresorcinols	0.5–1.5 g/kg bran; content influenced by rye genotype and environmental conditions	Antioxidant benefits; may bind proteins	(185–188)
β-Glucans	0.5–1.5% DM	↑ viscosity; health benefits (cholesterol-lowering)	(107, 112, 114)

Rye and barley have higher levels of trypsin inhibitors than oats and wheat, but compared to legumes, cereals have much lower amounts of inhibitors, particularly those affecting proteases and amylases; however, the presence of digestive enzyme inhibitors in cereals does not pose significant nutritional issues (105, 106). The adverse effects of trypsin inhibitors are mainly related to a reduction in the activity of digestive enzymes and a decrease in digestibility, as well as the utilization of protein, leading to poor nutrient utilization, potential pancreatic hypertrophy, and ultimately, reduced weight gain (111).

Among cereals, rye contains the most non-starch polysaccharides, which can lead to reduced intake, poor nutrient digestion, and ultimately lower body weight (112). The only effective method to neutralize their anti-nutritional effect is to use xylanases for the degradation of pentosans (113). It is noteworthy that rye contains higher levels of soluble arabinoxylans, compounds that benefit digestive health (41, 114). Furthermore, the antinutritional effect of water-soluble pentosans is weaker and may even benefit health by acting as prebiotics (115). Moreover, the inhibition of enzymes, such as α-amylases, may provide health benefits related to the prevention of T2D and obesity: the increased carbohydrate digestion time due to the enzyme inhibition decreases glucose absorption rate, and this affects the normal post-prandial plasma glucose level (116, 117).

In recent years, the incidence of cereal grain samples contaminated with ergot sclerotia and mycotoxins has increased worldwide (118–120) (Table 5). The increase in the incidence of contaminated samples may be associated with changes in the climate or agricultural practices. In the case of rye, the highest contamination levels were found in rye milling products, rye bread and rolls, and rye flakes, demonstrating that rye is the most contaminated among cereals (121).

**Table 5:** Mycotoxins in rye: typical levels and health impacts.

**Mycotoxin**	**Typical level (range/behavior)**	**Health impacts**	**References**
Ergot alkaloids (*Claviceps purpurea*)	Up to 1–5 mg/kg in contaminated grain (safe limit: < 0.5 mg/kg in the EU); higher than in wheat, oat (mean 594 μg/kg d.w.), and barley (below detection); rye = most susceptible crop; ↑ under high N fertilization and unfavorable weather	Vasoconstriction, neurotoxicity, reproductive disorders (“ergotism”)	(122, 185, 187, 189)
Deoxynivalenol (DON)	DON as marker; levels 50–2,000 μg/kg (EU limit 1,250 μg/kg); aat and barley—lower risk;s enriched in bran; mean 28.8 μg/kg; present in both organic and conventional rye	Nausea, vomiting, GI upset, and immune suppression	(118–120, 122, 128, 129, 190)
T-2/HT-2 toxins	Frequently co-occurs with DON 50–500 μg/kg; ~63% (T-2) and 57% (HT-2); mean 0.98–2.98 μg/kg; EU monitoring values used	Cytotoxic, hematotoxic, immunosuppressive	
Zearalenone (ZEN)	Frequently co-occurs with DON; levels 20–500 μg/kg; baking/extrusion ↓ < 25–80%; present in organic and conventional rye; also widespread in organic cereals	Estrogenic, endocrine disruption	
Ochratoxin A (OTA)	1–10 μg/kg in stored rye; post-harvest issue; extrusion ↓ ≤ 40%; baking ↓ < 30%	Nephrotoxic, carcinogenic (IARC 2B)	

In Europe, the ergot alkaloids (EA) producing fungus *Claviceps purpurea* is the most widespread *Claviceps* species that contaminates food supplies (122). The main crops affected by EAs are rye, barley, wheat, millet, oats, and triticale, with rye being the most sensitive to ergot alkaloids. It is highly susceptible to fungal growth when stored above 14% moisture and at temperatures of 18–30 °C (123). Specifically, EA concentrations in contaminated grain can increase or decrease after long-term storage (124). The alkaloids act on the nervous and vascular systems, causing ergotism (125).

Mycotoxins are toxic compounds produced by certain fungi on grains, such as rye, particularly in warm, humid conditions (122). Deoxynivalenol (DON), commonly produced by *Fusarium* species during improper storage or wet growing seasons, can cause nausea, vomiting, and feed refusal in livestock and humans (126). Zearalenone (ZEA), another mycotoxin from *Fusarium* species, mimics estrogen and disrupts hormonal balance, potentially causing reproductive issues in humans and animals (127). T-2 and HT-2 toxins produced by various *Fusarium* species are characterized as highly toxic and can damage the immune system, skin, and gastrointestinal tract (195). Notably, rye is the most resistant to *Fusarium* head blight and has the least kernel damage compared to triticale, durum, and soft wheat (128). A study of 60 winter rye samples from four varieties cultivated in three consecutive growing seasons across five different regions of Poland revealed the presence of DON, T-2 toxin, HT-2 toxin, and ZEA. Still, their concentrations were low, and none of the analyzed rye samples exceeded the maximum acceptable mycotoxin levels (129).

Although certain harmful agents can be present in rye, it's essential to carry out more in-depth and broad-ranging investigations to correctly identify the precise amounts of these agents and the potential risks they could entail, as the current research seems to show they are not likely to pose major dangers to human wellbeing when consumed in typical servings.

### 5.2 Methods to reduce antinutrients and toxins in rye products

**Figure 3:**
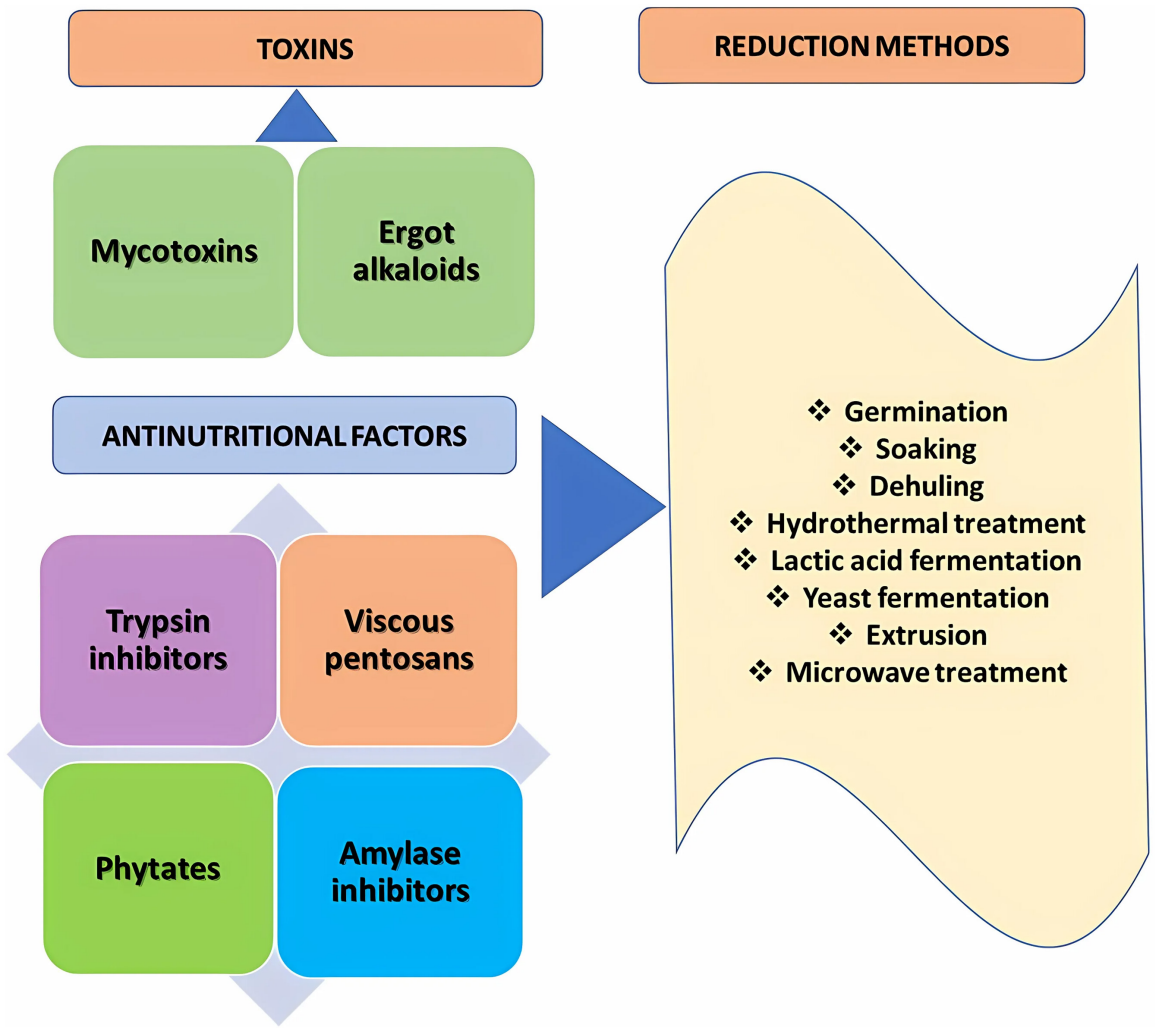
Conventional methods for reducing toxins and antinutrients in rye grain and rye products.

Various processing methods, such as soaking, germination, cooking, fermentation, and enzymatic treatment, can reduce or eliminate antinutritional components in cereals as well as in rye (105, 106) (Figure 3). In addition, several other methods have been proposed recently, including extrusion, microwave, and high-pressure processing (105, 130).

Germination effectively reduces phytate content in wheat, rye, and barley by 95–99%, as active phytase enzymes break down phytate salts, providing essential phosphate for the seedling (108). Rye has the highest phytase activity among grains, surpassing wheat, barley, corn, and rice (131). The phytate content of rye grain can be significantly lowered during soaking (132) because phytates are water-soluble (133).

Moreover, fermentation has been demonstrated to be an effective pre-treatment tool for wheat and rye to degrading antinutritive factors such as phytates and increasing mineral bioavailability (134). Sourdough lactic acid bacteria (LAB) can be used as a source of phytases, where fermentation leads to a more suitable pH for flour endogenous phytase activity (135). In addition to the nutritional benefits of the fermentation process, reductions in the levels of trypsin inhibitors and other antinutrients, as well as an increase in antioxidant capacity, have been reported during fermentation (136, 137). In addition, fermentation of sprouted rye also significantly increases the levels of folate, free phenolic acids, lignans, total phenolic compounds, and alkylresorcinols compared with natural rye (138).

Wet extrusion also offers advantages, including reducing ANs, increasing soluble dietary fiber, reducing lipid oxidation, and gelatinization of starch (105). Due to the high content of water-soluble pentosans in rye grains and, therefore, their high viscosity, they are of limited use in livestock feed (41). Studies have shown that extrusion significantly reduces the content of the main anti-nutrient of rye grain—water-soluble pentosans (41, 139).

Extrusion can be used as a tool to modify DF viscosity and starch retrogradation (139). Breaking down DF structure (140), which makes non-starchy polysaccharides more accessible to xylanases and increases the yield of fermentable oligosaccharides, can alter gut microbiota composition (141). As a result of extrusion processing, the content of water-soluble pentosans in the winter rye grain can be decreased by 1.34 times, leading to a certain decrease in starch in winter rye grain (41).

Extrusion can be used for a significant reduction of the ANF in cereal bran (reducing PA content by 54.51%, oxalates by 36.84%, and trypsin inhibitor by 72.39%) (142).

Microwave treatment also lowers antinutritional compounds in rye grain and significantly decreases the amount of water-soluble pentosans (41). Depending on the power and duration of the microwave treatment, the content of water-soluble pentosans can be decreased by up to 0.44%, resulting in a 2.4 times reduction in the viscosity of the aqueous extract (41). Overall, these various and diverse processing techniques, when employed effectively, significantly minimize the presence of antinutritional factors found in rye, thereby greatly enhancing its overall nutritional value and increasing its potential health benefits for those who include it in their diets.

## 6 Nutritional and bioactive properties of rye-based products

Rye flour with varying degrees of milling is widely used, especially in Eastern Europe, to produce soft breads and crispbreads using conventional or sourdough processes (6, 15).

**Figure 4:**
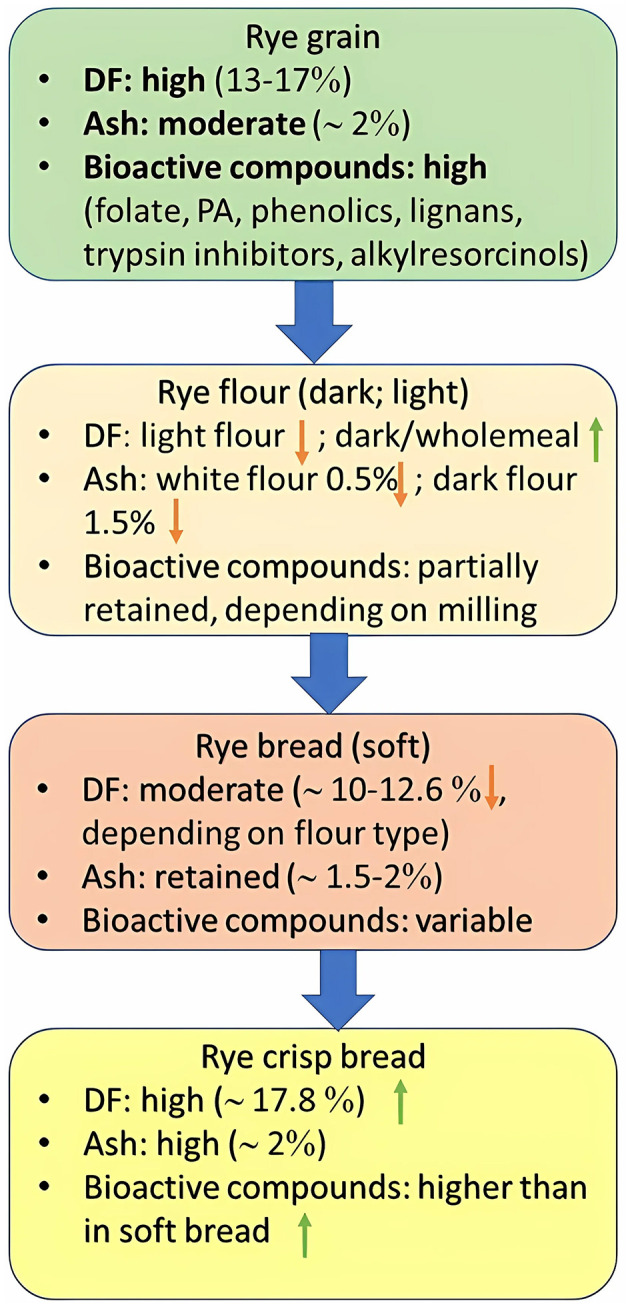
Nutrition quality of rye and rye products.

In Central Europe, white flour has ~0.5% ash, dark flour ~1.5%, and wholemeal up to ~2.0%. The baking industry commonly uses light rye flour, while dark rye flour is used for coarse dark breads. Whole-grain rye flour contains all grain components, which results in a coarser texture. Rye bread with a high proportion of whole-grain rye flour is typical in Eastern Europe (143), containing ~ 12.6% (soft bread) to ~ 17.8% (crispbread) total dietary fiber (26) (Figure 4).

The development of innovative whole-grain rye products is largely driven by the increasing consumer demand for high-quality foods rich in DF and bioactive compounds (144, 145). In response, the food industry is developing products with unique flavors, including confectionery items enriched with health-promoting components (146).

However, processing can have both beneficial and detrimental impacts on the nutrients and bioactive compounds in grains. In whole-grain processing, this may affect the bioavailability of bioactive compounds. In fermented, germinated rye, increases in folate, free PAs, total phenolics, lignans, and alkylresorcinols have been reported (147). Carbohydrate levels of rye bread can increase, while the total DF, β-glucan, and fructan contents may decrease when yeast fermentation and extrusion are used (148).

The starch hydrolysis rate and post-prandial glucose response of dense foods (e.g., pasta) are lower than those of white bread (149). Fermentation enhances starch hydrolysis, but sourdough acids can reduce the rate of gastric emptying (150). Prolonged sourdough fermentation alters the metabolite profile of whole-grain rye compared with milder whole-grain wheat, significantly increasing branched-chain amino acids (BCAAs) and their metabolites, microbial metabolites of phenolic acids, and other potentially bioactive compounds (6).

Fermentation, extrusion, and sourdough methods play a key role in shaping the nutritional benefits and bioactive-compound profile of rye products. While certain processes enhance the bioavailability of compounds like phenolics, BCAAs, and folates, others may reduce dietary fiber and modify carbohydrate levels. Understanding these implications is essential for developing rye-based foods that maximize health benefits while maintaining desirable sensory attributes. Ongoing research and innovation processes are crucial to enhancing rye products and benefiting consumer health and acceptance.

## 7 Environmental and socio-economic aspects of rye cultivation and consumption

### 7.1 Sustainability and environmental impact

**Figure 5:**
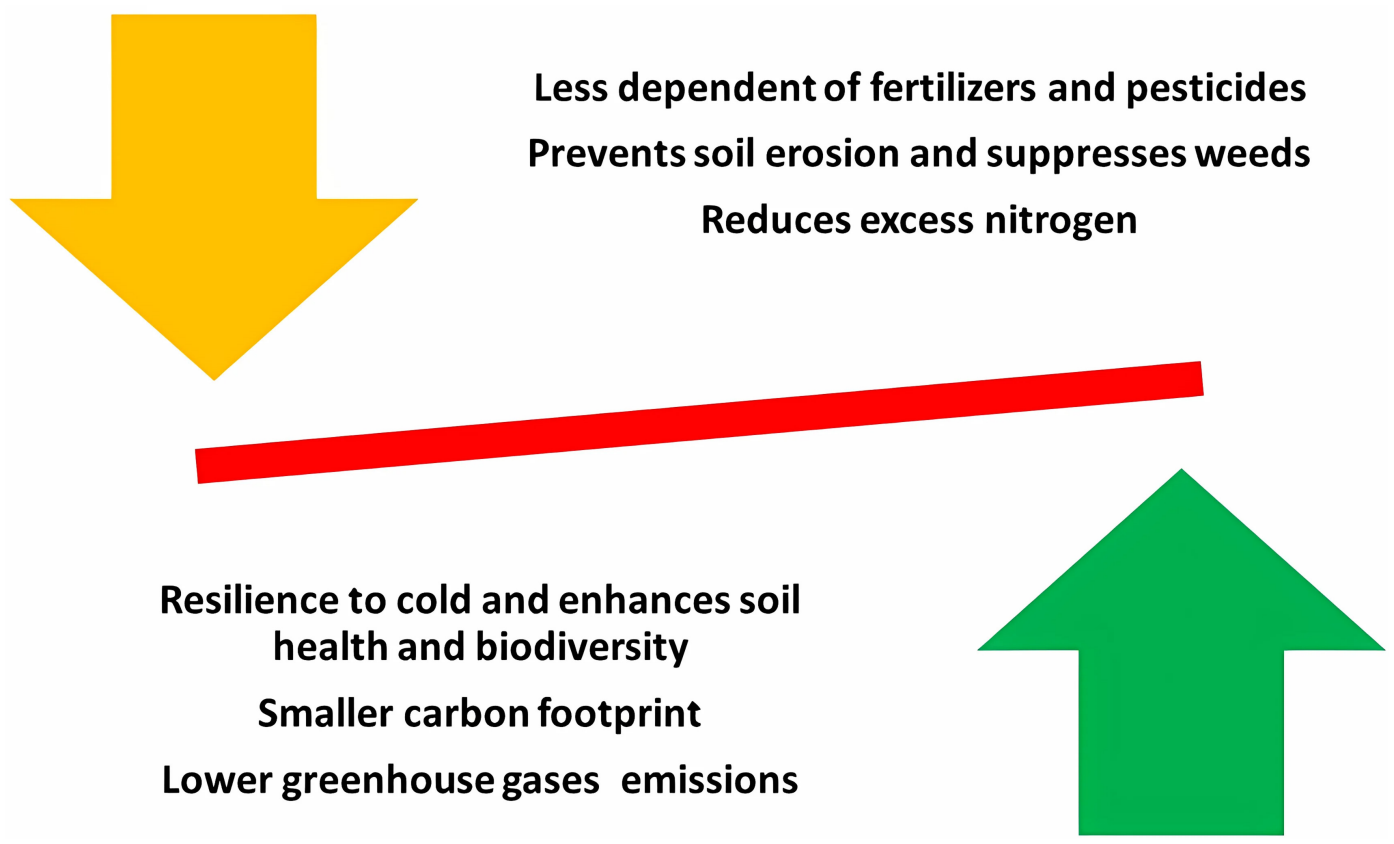
Advantages of rye to other cereals in sustainable agriculture strategies.

Addressing food security in the face of climate change requires transformative approaches that integrate human health and environmental sustainability (151). Advantages of rye over other cereals in sustainable agriculture strategies are presented in Figure 5. Rye offers a promising solution, particularly in northern Europe, where its resilience to cold and poor soils has historically outperformed wheat and barley (152). Recent studies have shown that rye emits ~20% fewer greenhouse gases and has a carbon footprint that is ~8% smaller compared to wheat, reinforcing its role in climate-friendly agriculture (153). Boosting rye production aligns with EU goals for a sustainable, low-emission future, and improving rye breeding is key to increasing its viability in contemporary farming.

Climate change has increased interest in more resilient, improved varieties (including hybrid rye) (154). Rye requires fewer fertilizers and pesticides than other cereals, making it a low-input crop that enhances soil health and biodiversity. As a winter cover crop, rye can help prevent soil erosion, suppress weeds, and improve soil quality (155). Moreover, double-cropping with winter rye reduces excess nitrogen, promoting sustainable intensification of agriculture (156). In summary, rye's environmental resilience, low input requirements, and multiple soil health benefits make it a vital crop for advancing sustainable agriculture and addressing the challenges of climate change.

### 7.2 Economic and social aspects

Rye has been cultivated for many thousands of years and is well-known for its cold resistance and ability to grow in low-fertility soil. Today, rye is integrated into grain production systems, mainly within the North German Plain, extending to Poland, Ukraine, Belarus, Scandinavia, and the Baltic countries. Whereas, the world average annual consumption of rye as food is only 1 kg per capita, it ranges from over 30–35 kg per capita in Poland, Lithuania, and Estonia to 10–15 kg per capita in Finland, Denmark, Sweden, and Germany (12, 15).

Winter rye plays a significant role in the economies and food cultures of countries where it is cultivated on over 90 thousand hectares, including Belarus, Denmark, Germany, Poland, Spain, and Ukraine (12). In recent years, its cultivation has also expanded in countries like China, Canada, and the United States (12).

Rye cultivation practices reduce dependence on high-impact animal protein production, thereby supporting global initiatives to remain within planetary boundaries (157), contributing to both environmental protection and healthier dietary patterns in line with international sustainability goals. Due to its unique phytochemical composition and high cultural significance in traditional foods, such as artisan bread and crackers, rye is also becoming attractive to health-conscious consumers who are preserving culinary traditions (7).

Nowadays, especially in Nordic countries, in addition to regular bread and bakery products, various food products made from rye (crisps, snacks, porridges, breakfast cereals, etc.) can already be found on the market, with the number of these products is constantly growing (158). New rye products are developed with diverse objectives. The food industry is seeking to develop new rye-based products, such as breakfast cereals, cracker chips, beverages, and snacks. These innovations expanded the assortment of rye products and attracted consumers seeking novel healthy foods (20). As consumer awareness of healthy eating increases, so does the demand for healthier products with higher dietary fiber and bioactive compound content. For this reason, new rye milling products are being developed (159) and rye baked goods enriched with fiber and bioactive compounds (160, 161). An innovative solution for developing new rye-based products is the application of extrusion-based 3D printing techniques to produce whole-grain flour-based snacks (162). In addition, in recent years, the possibilities of using rye products to produce higher-nutritional-value gluten-free baked goods have been widely explored (163).

### 7.3 Challenges and opportunities of a rye whole-grain diet

Despite their numerous benefits, whole grains face challenges, such as lengthy production times, perceived digestive issues, and competition from refined-grain products. Advanced processing techniques improve the digestibility and sensory quality of food, making these crops more accessible to a wider society (7, 164). Cultural attachment to meat, limited culinary knowledge, and concerns about affordability further hinder their widespread use (165). The development of affordable, innovative products and the dissemination of information to the wider public can increase their attractiveness and lead to greater integration in diets (166, 167).

Food intolerance is now being diagnosed in an increasing share of the population (168), making it difficult to adopt a balanced and diverse diet. In recent years, much attention has been paid to the development of higher-nutritional-value gluten-free products (169). Whole-grain rye products can be used to produce gluten-free bakery products by using a sourdough treated with specific peptidases that break down the gluten proteins, allowing the gluten-free claim (163). During sourdough fermentation, gluten proteins are broken down into harmless fragments. However, the degradation of toxic peptides during sourdough fermentation is often incomplete, and residual peptides are sufficient to trigger deleterious effects on people with CD (170). Moreover, standardization of the fermentation procedure is also challenging during production due to the microbiological variabilities in sourdough (171).

Concerning the conditions of the fermentation, some studies presented promising results of mixtures of probiotic LAB strains and long-term fermentation for decreasing contamination risk in gluten-free food (172). Mixed cultures of lactic acid bacteria in sourdough were shown to be more effective in reducing gluten and their toxic peptides than monocultures; furthermore, the addition of fungal proteases during sourdough improves gluten degradation, reaching < 20 mg/kg (173, 174). Fungal food-grade proteases from *Aspergillus oryzae* and *Aspergillus niger* gave rather promising results for the complete elimination of gluten from wheat-based products. However, the elimination of gluten proteins has technological disadvantages, as the formation of the gluten network is essential for baking quality. Therefore, the targeted degradation of toxic epitopes would be an optimal solution for the future (175). Rye products produced in this manner can increase the choice of high-quality gluten-free food options for consumers.

Demographic analyses reveal that younger urban populations are more receptive to the paradigms of a plant-based diet, which highlights the importance of targeted communication strategies to increase the adoption of healthy diets (164, 176). Ready-to-consume cereal-based products and protein-enriched rye foods are convenient to use, making these nutrient-rich products suitable for time-constrained modern consumers (177).

### 7.4 The role of policy and culinary education

Political action is essential to integrate target food crops into global dietary systems. Policies that combine traditional knowledge with new concepts can improve the visibility and accessibility of sustainable foods (178). Integrating relevant environmental narratives into policy and education initiatives can improve public understanding. By placing dietary transitions in a broader ecological and health context, policymakers can more effectively stimulate consumer behavioral changes (179).

Today, most rye is consumed as sifted flour with variable extraction rates across different Scandinavian countries. Rye is mostly consumed as sifted flour in Scandinavia, and its extraction rates affect the amount of fiber and other compounds retained (17). For example, Denmark offers two types of sifted rye flour (88% and 80%), Sweden has 80%, and Norway has 75% (17). In population studies, it is important to consider this fact when comparing health effects after intake of refined cereal products vs. whole-grain foods.

Professional culinary education programs that incorporate rye products into institutional and commercial food preparation can further promote these dietary alternatives. Engaging food professionals and businesses is a critical strategy for sustainable food choices and integration (180). Policies that encourage reduced consumption of animal products, combined with consumer education and promotion of plant-based food alternatives, are critical to addressing nutritional and environmental concerns. Integrating environmental and health considerations into campaigns can enhance consumer receptivity and drive meaningful change (177, 181).

Effective policy measures and targeted educational initiatives are essential to increase the visibility and consumption of rye as a sustainable and nutritious food source. By combining traditional knowledge with modern environmental and health narratives, policymakers can better motivate consumers to adopt plant-based diets that include rye products. Additionally, integrating rye into food industry practices will help normalize its use and expand its presence in institutional and commercial settings. Together, these efforts can foster meaningful dietary shifts that enhance both human health and environmental sustainability.

## 8 Conclusion

Rye is a highly versatile and sustainable cereal crop with exceptional nutritional, ecological, and economic value. Integrating rye alongside protein-rich legumes, such as peas, beans, and chickpeas, into sustainable food production systems could contribute significantly to global goals of reducing greenhouse gas emissions and improving dietary sustainability.

Rye and rye-derived products already play a significant role in cereal-based diets across Europe due to their high content of dietary fiber, protein, bioactive compounds, and essential micronutrients. Compared with wheat, rye offers a more balanced nutrient profile. However, its gluten content remains a barrier for people with celiac disease. This contrast highlights both the strengths and the limitations of rye as a dietary staple.

From a nutritional perspective, rye is abundant in dietary fiber, vital minerals, and bioactive compounds. It promotes digestive health, helps stabilize blood sugar levels, and supports bone health. From an ecological perspective, its ability to thrive in challenging growing environments with relatively low ecological impact makes rye an ideal candidate for sustainable agriculture. When combined with legumes, it can further enhance biodiversity and contribute to mitigating climate change.

Beyond its nutritional and ecological benefits, the successful integration of rye into future food systems will depend on the implementation of supportive strategies at the societal level. Robust policy initiatives that combine traditional agricultural practices with contemporary sustainability objectives, along with nutritional and culinary education for both industry professionals and consumers, are essential. These measures can raise awareness, strengthen consumer acceptance, and encourage healthier dietary changes.

In summary, these factors underscore rye's vital role in fostering resilient, nutritious, and environmentally sustainable food systems that support both public health goals and ecological responsibility.

## 9 Future directions

Future directions for rye research and development include exploring novel processing methods to enhance the technological properties of rye, developing more appealing rye-based products to increase consumer acceptance, improving their nutritional density, investigating the mechanisms underlying health benefits, and promoting sustainable cultivation through diversified crop rotations to enhance grain quality.

Another priority is making rye-based products more suitable for people with celiac disease and gluten intolerance. Fermentation and enzyme-based processing also hold promise for producing gluten-reduced or gluten-free rye foods without compromising their nutritional value.

Equally important are advances in processing technologies. Innovative approaches such as controlled fermentation, enzymatic hydrolysis, and improved milling techniques can enhance dough rheology, baking performance, and the release of bioactive compounds. These technological improvements will not only support the production of healthier bread and bakery products but also pave the way for applications in feed, biomaterials, and pharmaceuticals.

Ongoing improvements in breeding, processing, and product development should be matched with efforts to meet changing consumer expectations. The growing demand for functional, sustainable, and health-promoting foods highlights the need for rye-based innovations that strike a balance between sensory quality and nutritional benefits. This creates opportunities for both traditional products, such as dark breads, and novel offerings, including gluten-free snacks and functional foods enriched with bioactive compounds.

Finally, policy frameworks and educational initiatives will be critical for ensuring the widespread adoption of rye in global food systems. Supportive regulations, incentives for sustainable farming practices, and awareness campaigns can help integrate rye into mainstream diets. Nutritional education for consumers and training for food industry professionals can further increase acceptance and demand, ensuring that the benefits of rye are fully realized in future food systems.
